# Environmental behaviors and ecotoxicity of imidaclothiz: a comprehensive review on fate, degradation processes, and ecological risks

**DOI:** 10.3389/fmicb.2025.1724551

**Published:** 2026-01-13

**Authors:** Ju Yang, Hao Chen, Ke-Wei Song, Yi-Fei Chen, Yue Shen, Wen-Jing Li, Xiao-Fang Li, Peng-Fei Zhai, Yun-Xiu Zhao

**Affiliations:** 1 College of Marine and Biological Engineering, Yancheng Teachers University, Yancheng, China; 2 Jiangsu Key Laboratory for Bioresources of Saline Soils, Jiangsu Synthetic Innovation Center for Coastal Bio-Agriculture, College of Wetlands, Yancheng Teachers University, Yancheng, China; 3 Shandong Provincial University Laboratory for Protected Horticulture, Shandong Facility Horticulture Bioengineering Research Center, Weifang University of Science and Technology, Weifang, China

**Keywords:** degradation mechanism, ecological risk, environmental fate, imidaclothiz, neonicotinoid insecticide

## Abstract

Neonicotinoid insecticides (NEOs) are among the most widely used pesticide classes globally, primarily applied to control crop pests and vector plant pathogens. As a fourth-generation NEO, imidaclothiz (IMT) has seen rapidly expanding agricultural use across Asia—particularly in China—in recent years. However, increasing evidence highlights the persistence of NEO residues in the environment and their toxic effects on non-target organisms. The frequent detection of NEOs in aquatic systems indicates growing contamination that may pose significant risks to human and ecosystem health. Microbial degradation, alongside chemical pathways, plays a crucial role in mitigating the environmental impacts of these pesticides, with microbial remediation emerging as a promising ecological strategy. Nevertheless, current research on IMT remains limited: only a few IMT-degrading microorganisms and key metabolic enzymes (e.g., cytochrome P450s, manganese peroxidases) have been identified, and systematic mechanistic studies are largely lacking. Moreover, the ecotoxicity effects of IMT and its environmental transformation products—particularly their broader ecological implications—are poorly synthesized and insufficiently understood. This review provides a comprehensive assessment of the environmental behavior, degradation mechanisms, and ecotoxicity impacts of IMT, offering critical insights into the multiscale environmental risks of NEOs and supporting future ecological risk assessments and sustainable pesticide management.

## Graphical abstract

**Figure:**
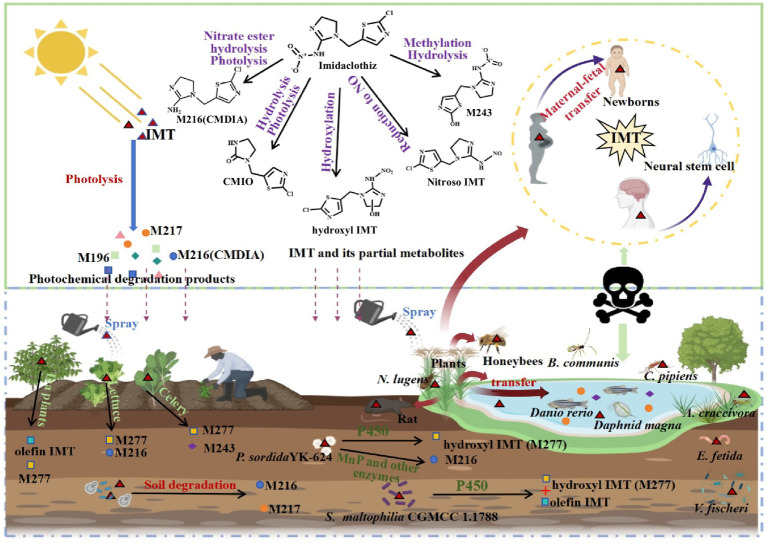

## 1 Introduction

Since their commercialization in the early 1990s, neonicotinoid insecticides (NEOs) have revolutionized agricultural pest management and steadily come to dominate the global insecticide market. In 2024, worldwide NEO sales reached USD 5.593 billion, with projections estimating growth to USD 9.1 billion by 2034, driven by a compound annual growth rate (CAGR) of 5.6% (Global Market Insights, 2025). Among major producers and users, China—owing to its large agricultural footprint and strong innovation capacity—plays a pivotal role in the development, manufacture, and application of NEOs (Liao et al., 2024). Imidaclothiz (IMT), a fourth-generation NEO independently developed by Jiangshan Agrochemical Co., Ltd. (Nantong, China), represents a significant advancement within this insecticide class. Chemically designated as 1-[(2-chloro-5-thiazolyl)methyl]-4,5-dihydro-N-nitro-1H-imidazol-2-amine (Wu et al., 2010a), IMT exerts its insecticidal activity by selectively binding to insect nicotinic acetylcholine receptors (nAChRs), thereby disrupting synaptic transmission—similar to earlier NEOs such as imidacloprid (IMI) (Houchat et al., 2020; Tao et al., 2021). IMT is characterized by high systemicity, rapid uptake, and strong contact toxicity (Jiang et al., 2022), and is widely used to control piercing-sucking pests across diverse crops, including rice, wheat, vegetables, tobacco, cotton, fruit trees, and tea (Li et al., 2014). In addition, it has demonstrated notable efficacy against multiple insect orders, particularly rice stem borers such as *Chilo suppressalis* and *Scirpophaga incertulas* (Pang et al., 2020; Wu et al., 2010a; Lewis et al., 2016; Nidheesh et al., 2020).

Despite the agronomic advantages of IMT and its NEO analogues—such as broad-spectrum activity, persistence, and high systemicity—their widespread use has raised significant environmental concerns. Their high aqueous solubility and mobility contribute to frequent environmental detection, with increasing reports of contamination in soils, surface waters, and groundwater (Hladik et al., 2018; Klingelhöfer et al., 2022). As a result, fourth-generation NEOs like IMT are becoming increasingly ubiquitous in agricultural regions, particularly in Asia (Wu et al., 2010b; Shao et al., 2013). However, compared with legacy NEOs such as IMI or thiamethoxam (TMX), knowledge on IMT’s environmental behaviors, degradation pathways, and ecotoxicity impacts remains insufficiently developed (Ma et al., 2021).

Emerging evidence suggests that IMT poses ecological risks to a range of non-target organisms, including pollinators, aquatic invertebrates, and microbes (Ma et al., 2021; Wang Z. et al., 2023; Wang J. et al., 2023; Zhang et al., 2024). Like other NEOs, IMT may disrupt pollinator foraging and navigation behavior, potentially destabilizing pollination networks (Dussaubat et al., 2016; Wu-Smart and Spivak, 2016). Broad-spectrum ecotoxicity has also been observed, with adverse effects reported across aquatic and terrestrial ecosystems (Beketov et al., 2013; Alsafran et al., 2022; Zhao et al., 2025a). Despite its rapid expansion in use, systematic studies of IMT’s environmental fate remain scarce. Given its high solubility, IMT is readily absorbed by plants and translocated, raising concerns about its entry into food webs and potential risks to human health (Tao et al., 2021; Jiang et al., 2022). For example, in South China’s intensively farmed Zengcheng region, IMT and related compounds were detected in over 95% of 351 soil samples, with median concentrations in vegetable fields (23 ng/g) significantly exceeding those in rice paddies and orchards (Yu et al., 2021). In parallel, monitoring data revealed the presence of multiple NEO residues in over 90% of commercial milk samples from Chinese markets (Wei et al., 2023), and alarmingly, traces of NEOs and metabolites were detected in urine samples of over 90% of newborn infants, highlighting the pervasiveness of chronic low-level exposure (Tang et al., 2024).

Although some studies have reported abiotic and biotic degradation of IMT—including photolysis, hydrolysis, and microbial metabolism—the transformation kinetics and environmental toxicity of its degradation products remain largely unresolved (Ma et al., 2021; Todey et al., 2018). Under ultraviolet radiation, IMT may degrade to form nitroso or dechlorinated intermediates, some of which display greater toxicity than the parent compound (Ma et al., 2021). Soil bacteria can metabolize IMT via nitro-reduction or cleavage of the C–N bond, but the specific enzymatic mechanisms, including the role of nitroreductases, have yet to be fully elucidated (Ma et al., 2021; Liu et al., 2011). In addition, hydrolysis under acidic or alkaline conditions generates derivative compounds whose ecological risks remain poorly characterized (Ma et al., 2021; Todey et al., 2018; Liu et al., 2011). Thus, further research is urgently needed to clarify these complex environmental transformation processes and their ecological ramifications.

The sublethal and chronic toxic effects of IMT on non-target species have attracted increasing scientific and regulatory attention (Zhuang et al., 2024). For instance, exposure to environmentally relevant concentrations of IMT impairs bee neurophysiology and pollination behavior (Ma et al., 2021). IMT showed low acute toxicity to the *Daphnia magna* (48-h EC₅₀ > 10 mg a.i. L^−1^) and *Danio rerio* (96-h LC₅₀ > 10 mg a.i. L^−1^). However, Ecological Structure Activity Relationships (ECOSAR) predictions indicated its degradation products M149 and M217 pose significantly higher toxicity to *D. magna*, with acute levels approximately 79- and 10-fold greater than the parent compound, suggesting elevated ecological risks to freshwater ecosystems (Ma et al., 2021). In soil systems, IMT may inhibit key enzymes such as dehydrogenases and ureases, thereby disrupting microbial functions critical for carbon and nitrogen cycling (Mahapatra et al., 2017; Zhuang et al., 2024). However, most current environmental risk assessment (ERA) frameworks emphasize acute, high-dose exposures and largely overlook long-term, low-dose exposures, including oxidative stress, genotoxicity, and synergistic toxicity of metabolites (Zhuang et al., 2024; Yin et al., 2025). These omissions likely contribute to underestimations of ecological and human health risks.

While several comprehensive reviews have synthesized environmental and toxicological data for the NEO class as a whole, a dedicated, in-depth review focusing specifically on IMT is currently lacking. This review aims to fill this critical knowledge gap. By providing a compound-specific synthesis for IMT, this review elucidates its unique attributes, including its environmental behaviors, distinct degradation pathways and metabolites, as well as its particular toxicological profile. We evaluate the fate of IMT across environmental compartments (soil, water, and plants), detail known biotic and abiotic degradation mechanisms, and assess ecotoxicity at multiple biological levels—from molecular to ecosystem. By integrating fragmented findings into a cohesive framework, this review aims to inform future research, guide evidence-based regulation, and support the development of next-generation insecticides with improved environmental compatibility and degradability.

## 2 Environmental fate

### 2.1 Literature search strategy

A systematic literature search on IMT was performed using Web of Science, PubMed, and Google Scholar. Key search terms included “imidaclothiz,” “environmental fate,” “degradation,” “metabolite,” “ecotoxicology,” and “toxicity.” The search, encompassing publications up to 2025 with no start date limit, was primarily restricted to English and Chinese sources. The screening of retrieved articles and their reference lists ensured a comprehensive and transparent review.

### 2.2 Environmental behaviors of IMT in soil

NEOs exhibit high water solubility, a key property driving their systemic pesticidal action (García-Hernández et al., 2025). However, in seed treatments, only ~5% of applied NEOs are absorbed by crops, while the remaining 80–95% persist in the environment, where they readily migrate to soils, surface waters, and aquifers (Goulson, 2013; Smalling et al., 2017; Wood and Goulson, 2017). Global monitoring efforts have consistently documented NEO residues across environmental compartments including soil, sediment, and aquatic systems (Li et al., 2025; Cao et al., 2024).

**Figure 1:**
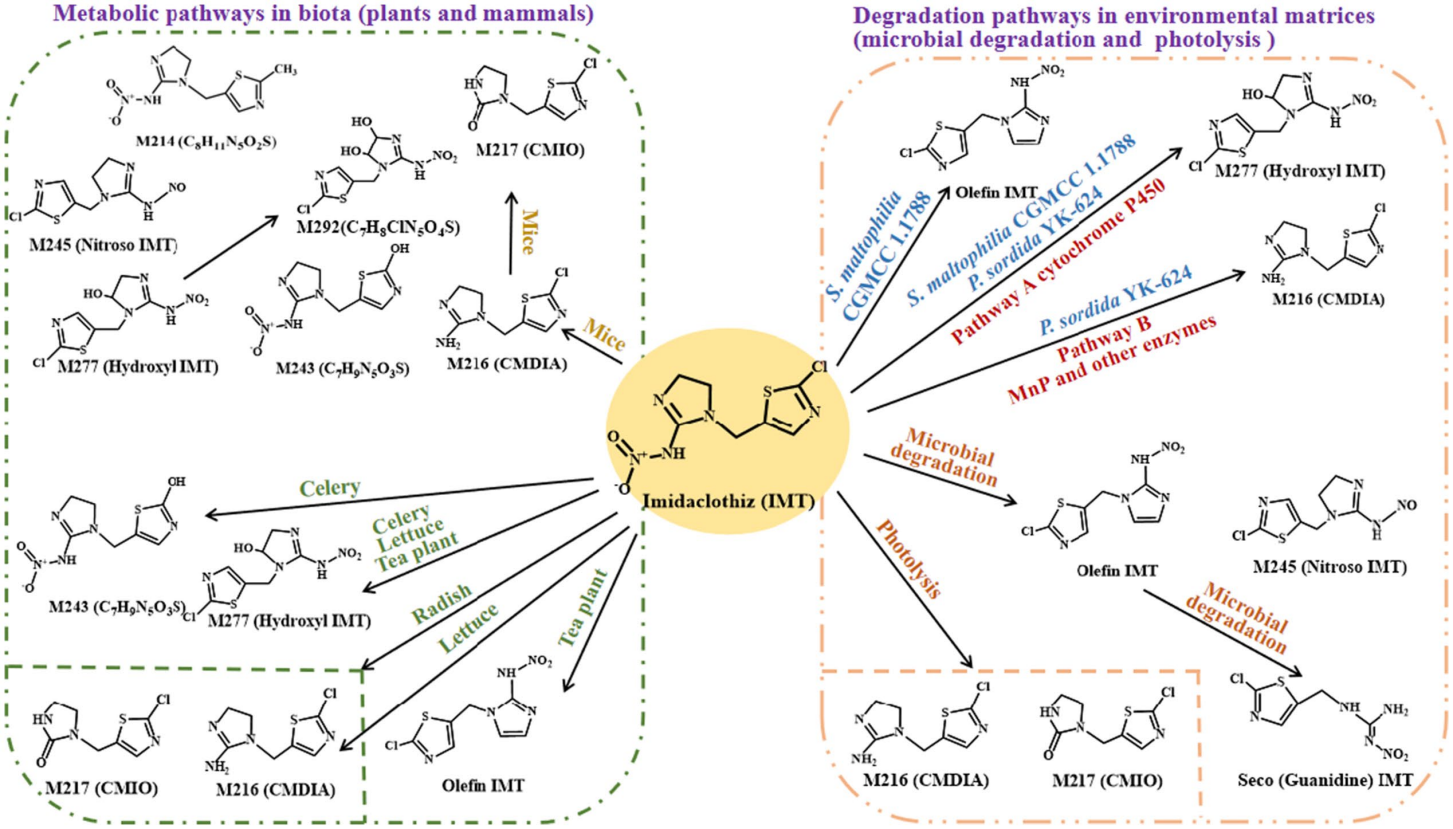
Proposed transformation pathways of IMT. The transformation of IMT proceeds via distinct pathways depending on the system: metabolic pathways in biota (plants and mammals) and degradation pathways in environmental matrices (primarily mediated by microorganisms in soil, or by abiotic processes such as photolysis in surface water). 1-((2-Chlorothiazol-5-yl)-methyl)-4,5-dihydro-1H-imidazol-2-amine (CMDIA), 1-((2-chlorothiazol-5-yl)methyl)imidazolidin-2-one (CMIO).

NEOs are notably persistent. For example, TMX and IMI display aqueous half-lives exceeding 800 and 4,000 days, respectively (Karmakar et al., 2009; Zheng and Liu, 1999). Similarly, IMT demonstrates strong environmental stability. In sterilized soil, only 9% of IMT was degraded within 25 days, with a half-life of 173.3 days. In contrast, in non-sterilized soil, 25.1% of IMT was degraded over a longer period of 25 days, exhibiting a shorter half-life of 57.8 days. The metabolites nitroso-IMT, olefin-IMT, and guanidine-IMT were detected (Figure 1) (Liu et al., 2011). Clearly, the degradation of IMT is primarily attributed to soil microbial activity (Liu et al., 2011; Yin et al., 2025). However, the observed degradation in sterile conditions suggests that a portion of IMT may also undergo chemical hydrolysis. In natural soils, IMT degradation is influenced by oxygen levels, temperature, soil type, and organic matter. Under anaerobic conditions in black soil, its half-life drops to 18.24 days (Ma et al., 2021). IMT degrades more rapidly with increased temperature and organic content, and while it undergoes hydrolysis and photolysis under alkaline conditions, it remains stable in acidic or neutral waters. NEO persistence is further modulated by soil adsorption, microbial activity, ambient pH, and humidity (Bonmatin et al., 2015; Zhang et al., 2018).

### 2.3 Environmental behaviors of IMT in aquatic systems

NEOs readily enter aquatic ecosystems via rainfall, irrigation runoff, and drainage networks, contributing to widespread contamination (Thuyet et al., 2012). Concentrations ranging from nanograms to milligrams per liter have been detected in diverse aquatic systems worldwide, raising ecological concern (Wang et al., 2019). These include rivers and lakes near Sydney (Australia), 38 river systems in the U. S., Japan’s Seto Inland Sea, and 36 major rivers in China’s Pearl River Basin, Bohai Rim, and Yellow Sea regions (Hladik and Kolpin, 2016; Hano et al., 2019; Xiong et al., 2018; Naumann et al., 2022). In Missouri, USA, 63% of sediment samples from a protected nature reserve contained detectable NEO residues (Kuechle et al., 2019). In China, concentrations in surface waters often exceed the chronic toxicity threshold (35 ng/L), with hotspots such as Beijing and the Qilu Lake basin surpassing acute toxicity benchmarks (200 ng/L) (Liao et al., 2024).

A meta-analysis of 43 studies across 10 countries found that NEOs are most prevalent in agricultural regions of East Asia and North America, with an average IMT concentration of 24.5 ± 2.9 ng/L (Tsegay et al., 2024). Environmental parameters strongly shape their distribution: concentrations increase with temperature, redox potential, and cropping intensity, and decrease with higher river flow, pH, dissolved oxygen, and precipitation (Wang Z. et al., 2023; Wang J. et al., 2023). Surveys in Jiangsu Province, China, revealed that IMT consistently exhibited the highest frequency and concentration among all detected NEOs in surface sediments (Huang et al., 2022). While agriculture continues to dominate as the main source of NEO emissions, their expanding application in urban greenspaces and domestic settings calls for more comprehensive risk assessments.

### 2.4 Behavior and metabolic transformation of IMT in plants

IMT, a structural analog of IMI, is a novel systemic NEO that has seen increasing agricultural application due to its high insecticidal potency (Du et al., 2023). Despite its widespread use, knowledge regarding IMT’s absorption, translocation, and metabolic fate in plants remains limited, constraining efforts to evaluate its environmental persistence and potential dietary exposure risks (Jiang et al., 2022; Tao et al., 2021). Ge et al. (2021) investigated the uptake and metabolic transformation of IMT in three representative vegetables—celery, lettuce, and radish—using a hydroponic system. In celery tissues, multiple IMT metabolites were identified, including hydroxyl-IMT, IMID-3 (M243B), IMID-4 (M243A), M216A, and M216B. Lettuce leaves similarly accumulated hydroxyl-IMT, M216A, and M216B. M216A, and M216B were accumulated in radish leaf (Figure 1) (Ge et al., 2021; Tao et al., 2021). These findings indicate that IMT is readily absorbed by roots and efficiently translocated to aerial parts via the transpiration stream, resulting in notable foliar accumulation and metablism (Tao et al., 2021).

Mass balance analyses revealed a marked decline in total IMT residues within the plant–water system over time, with losses ranging from 31.8 to 45.6% by the end of exposure, suggesting extensive in planta transformation. Metabolite profiling further identified five transformation products in celery, three in lettuce, and two in radish, implicating hydroxylation, nitrate ester hydrolysis, and methylation as the principal metabolic pathways (Tao et al., 2021). Additional studies have detected IMT metabolites, including hydroxyl-IMT and olefin-IMT, in tea plants, suggesting similar transformation dynamics across diverse plant species (Ge et al., 2021).

Collectively, these results highlight the dual role of plants as both passive reservoirs and active sites of metabolic transformation for NEOs like IMT, with significant implications for their environmental behaviors, persistence, and trophic transfer potential (Tao et al., 2021; Wu et al., 2014).

## 3 Degradation mechanism of IMT

### 3.1 Chemical degradation

Numerous studies have identified temperature and pH as critical environmental parameters regulating the hydrolysis of NEOs (Chen et al., 2018). At 25 °C, IMT exhibits pronounced hydrolytic stability, with hydrolysis rates remaining below 10% over 85 days across pH 4.0, 7.0, and 9.0 buffer solutions. However, at elevated temperatures (e.g., 50 °C), hydrolysis is markedly accelerated, particularly under alkaline conditions (pH 9.0), where the half-life shortens dramatically to 4.95 days (Ma et al., 2021). This temperature- and pH-dependent behavior aligns with that of other NEOs such as IMI and TMX, which also degrade more readily under alkaline and thermophilic conditions.

Regarding photodegradation, IMT follows typical first-order photolytic kinetics under xenon-lamp-simulated sunlight at 25 °C. The estimated half-lives are 16.50 h, 14.75 h, 2.52 h, and 2.67 h in pH 4.0, 7.0, and 9.0 buffers and ultrapure water, respectively (Ma et al., 2021). Under photolytic conditions, seven metabolites of IMT were identified via UHPLC-QTOF-MS analysis, with 1-((2-chlorothiazol-5-yl)- methyl)-4,5-dihydro-1H-imidazol-2-amine (CMDIA), 1-((2- chlorothiazol-5-yl)methyl)imidazolidin-2-one (CMIO), and M198 emerging as the predominant transformation products. Notably, CMDIA and CMIO were detected in both microbial degradation pathways and soil degradation pathways (Figure 1; Ma et al., 2021; Yin et al., 2025). Since soil degradation primarily results from microbial activity, this indicates a dual pathway formation: photolysis and microbially mediated degradation.

The above results indicate that photolysis is significantly enhanced under alkaline and low-ionic-strength conditions. This trend may result from pH-mediated modulation of hydroxyl radical (·OH) reactivity or from the inhibitory influence of dissolved metal cations on photodegradation. Similar behaviors have been reported for IMI and TMX, which are readily hydrolyzed in alkaline buffers but remain relatively stable in neutral environments (Zheng and Liu, 1999; Karmakar et al., 2009). Undoubtedly, most NEOs are more easily hydrolyzed in alkaline environments (Ma et al., 2021). Additionally, IMT demonstrates increased susceptibility to hydrolysis at higher temperatures, suggesting that climate-induced warming may accelerate its environmental dissipation (Todey et al., 2018). In summary, temperature and pH are key environmental drivers controlling the hydrolytic and photolytic breakdown of IMT. Elucidating these factors is essential for predicting its environmental persistence and refining ecological risk assessments of NEOs.

### 3.2 Microbial degradation

Previous study revealed that the bacterial strain *Stenotrophomonas maltophilia* CGMCC 1.1788 mediates the biotransformation of IMT into hydroxyl-IMT and olefin-IMT through parallel hydroxylation and dehydrogenation pathways (Zhao et al., 2014; Dai et al., 2010). Quantitative analysis showed that 36.2% of the initial IMT was converted to these two metabolites after 84 h of incubation. Importantly, when hydroxyl-IMT was supplied as the sole substrate, *S. maltophilia* CGMCC 1.1788 failed to catalyze its conversion to olefin-IMT, indicating distinct metabolic routes for these transformations (Figure 1; Table 1) (Dai et al., 2010). Interestingly, the substitution of the 6-chloropyridinyl ring in IMT for the more common 2-chlorothiazolyl group (found in other NEOs) does not significantly alter either the hydroxylation rate or the enzymatic target sites. The use of piperonyl butoxide (PBO), a known cytochrome P450 monooxygenase (CYP) inhibitor, substantially suppressed hydroxylation activity, implicating CYPs as the primary enzymes mediating IMT biotransformation (Zhao et al., 2014; Dai et al., 2010). In non-sterilized natural soils, IMT exhibits limited degradation, with only 25.1% reduction observed over 25 days, indicating considerable environmental persistence. Microbial transformation of IMT primarily yields olefin, nitroso, and guanidine derivatives, resembling the degradation pathways of IMI, particularly its nitroso-guanidine intermediates (Liu et al., 2011). These findings underscore the pivotal role of indigenous microbial communities or bacteria in pesticide dissipation and provide a theoretical foundation for environmental risk evaluation and bioremediation strategies.

**Table 1:** The ability of isolated microorganisms in degrading IMT.

Target NEO	Microorganism	Time/Concent	Degradation (%)	Products	References
IMT	*Stenotrophomonas maltophilia* CGMCC 1.1788	3.5 days/ ≈ 600 μM	36.2	5-hydroxy-IMT and olefin-IMT	Dai et al. (2010)
IMT	*Pseudomonas monteilii* FC02	14 days/≈ 0.382 μM	15.09%	Not mentioned	Cai et al. (2022)
IMT	*Phanerochaete sordida* YK-624	20 days/10 μM	47.5	Olefin-IMT, CMDIA and CMIO	Yin et al. (2025)
IMT	*Phlebia acerina* S-LWZ20190614-6	20 days/10 μM	36.4	Not mentioned	Yin et al. (2025)
IMT	*Irpex lacteus*	20 days/10 μM	38.4	Not mentioned	Yin et al. (2025)
IMT	*Trametes versicolor*	20 days/10 μM	21.8	Not mentioned	Yin et al. (2025)

In addition to these bacterial pathways, plant growth-promoting bacteria (PGPB) have demonstrated significant potential in phytoremediation systems (Cai et al., 2022). For instance, *Pseudomonas monteilii* FC02, isolated from pesticide-contaminated agricultural soils, significantly enhanced both duckweed biomass and NEO uptake in a plant-microbe co-remediation system. IMT removal was limited to 15.09% with bacteria alone after 14 days (Table 1). However, a remarkable 96.42% removal was achieved with duckweed inoculation, highlighting the highest NEO clearance. This confirms the strain’s strong potential in plant-assisted remediation (Cai et al., 2022).

Beyond bacterial contributions, four types of fungi also exhibit strong IMT-degrading capabilities. After 20 days of incubation, the removal efficiencies of IMT by the fungi *Trametes versicolor*, *Irpex lacteus*, and *Phlebia acerina* S-LWZ20190614-6 reached 21.8, 38.4, and 36.4%, respectively (Table 1). Under identical degradation conditions, the white-rot fungi *Phanerochaete sordida* YK-624 exhibited superior degradation performance, achieving a 47.5% IMT degradation rate within the same 20-day period (Table 1). Mechanistic studies revealed that both CYP and manganese peroxidases (MnP) enzymes are key mediators in IMT metabolism. CYPs catalyze the hydroxylation of IMT to 5-hydroxy-IMT, which is then dehydrated to olefin IMT. A parallel nitro-reduction pathway involves hydrolysis of IMT to CMDIA, followed by deamination and oxidation to CMIO (Figure 1). Enzyme activity assays and inhibitor experiments confirmed the central roles of CYP and MnP enzymes in these pathways (Dai et al., 2010; Yin et al., 2025).

In conclusion, microbial transformation of IMT and related compounds involves synergistic metabolic pathways mediated by both bacterial CYPs and fungal MnP systems. However, it should be noted that other metabolic pathways may also contribute to IMT degradation, which requires further investigation. These findings are crucial for advancing the understanding of NEO environmental dissipation and for guiding the development of effective microbially mediated remediation technologies.

## 4 Toxic effects of IMT

The metabolic fate and ecological impact of pesticide transformation products remain poorly understood. Emerging evidence suggests that certain NEO metabolites may exhibit greater ecotoxicity than their parent compounds, necessitating heightened environmental scrutiny (Liu et al., 2024). IMT, a novel NEO bearing a thiazolyl chlorine substituent, an imidazolidine ring, and an N-nitro functional group (=NNO–NO₂), is structurally analogous to widely used compounds such as IMI, TMX, and clothianidin (CLO) (Casida, 2011). IMT exhibits relatively long half-lives and produces multiple degradates (e.g., M216, M217, M198), some of which show higher aquatic toxicity than the parent compound (Ma et al., 2021). In comparison, IMI is generally moderately to highly persistent and forms characteristic metabolites such as 6-chloronicotinic acid, olefin, and desnitro derivatives. TMX degrades more rapidly in certain matrices but is readily converted to CLO, making its environmental persistence and risk largely CLO-driven. CLO itself is among the most persistent NEOs yielding stable thiazole fragments such as 2-chloro-5-methy-thiazole (CMT) and methyl 3-(thiazole-5-yl) methyl guanidine (TMG) with notable persistence and ecotoxicity (Casida, 2011; Dai et al., 2010; Zhao et al., 2025b). Overall, lMT and CLO, both bearing a thiazolyl substituent, tend to formstable thiazole-ring metabolites that may increase residue longevity and ecological risk; however this structural effect is not determinative, as overall molecular features (e.g., nitroguanidine vs. cyanoamidine groups) and environmental factors jointly shape fate and toxicity. Given the high environmental persistence, intricate degradation pathways, and potent toxicity of these compounds and their metabolites, a systematic evaluation of IMT’s environmental behaviors and toxicological profiles is imperative.

### 4.1 Ecotoxicity to aquatic organisms

For aquatic organisms, laboratory experiments demonstrated that IMT exhibits low acute toxicity to both *D. magna* and *D. rerio*. The measured 48-h EC₅₀ for *D. magna* was 12.80 mg a.i. L^−1^, which is substantially lower than the predicted value of 9923.43 mg a.i. L^−1^ (Table 2; Ma et al., 2021). Yin et al. (2025) predicted the acute and chronic toxicity of IMT and its metabolites in various aquatic organisms using the ECOSAR program. IMT exhibits low toxicity to green algae (EC₅₀ = 427 mg L^−1^) and low acute toxicity to fish (LC₅₀ = 19 mg L^−1^), but shows high toxicity to *D. magna* (LC₅₀ = 0.323 mg L^−1^). However, this prediction contrasts with experimental measurements by Ma et al. (2021), which classified IMT as having low toxicity to *D. magna* (EC₅₀ = 12.80 mg a.i. L^−1^). This discrepancy highlights the variation between software predictions and experimental measurements. Nevertheless, both lines of evidence indicate that IMT may pose potential ecological risks to aquatic organisms (Ma et al., 2021).

**Table 2:** Toxic effects of IMT and its metabolites on soil organism, aquatic organisms, insects, and mammals.

Category	Specie	Compound	Dose/treatment	Effect	References
Soil organism	*E. fetida*	IMT	28 d exposure to IMT (0.3 or 1.0 mg kg^−1^) followed by 28 d incubation in clean soil.	Inhibited GST, CE, CAT, POD; SOD unaffected. POD remained suppressed through day 35; Oxidative/DNA damage (↑ OTM).	Zhang et al. (2017)
Insect	*B. communis*	IMT	Sublethal exposure (LC₁₀: 11.48 mg·L^−1^; LC₃₀: 28.03 mg·L^−1^)	Reduced survival, parasitism, and longevity (F1 generation); extended developmental duration. Transgenerational stress inheritance. Disrupted energy metabolism, detoxification pathways, gut microbiota, and gene expression.	Du et al. (2023)
	*A. craccivora*	IMT	LC_50_ = 0.15 mg·L^−1^ (72 h)	High insecticidal activity	Dai et al. (2010)
	*A. craccivora*	5-hydroxy-IMT	LC_50_ = 0.67 mg·L^−1^ (72 h)	Retains high efficacy (comparable to IMT).	Dai et al. (2010)
	*A. craccivora*	Olefin-IMT	LC_50_ = 0.29 mg·L^−1^ (72 h)	Retains high efficacy.	Dai et al. (2010)
	*N. lugens*	IMT	LC_50_ = 0.77 mg·L^−1^ (96 h)	Highly effective.	Dai et al. (2010)
	*N. lugens*	5-hydroxy-IMT	LC_50_ > 4 mg·L^−1^ (96 h)	Negligible activity.	Dai et al. (2010)
	*N. lugens*	Olefin-IMT	LC_50_ = 1.63 mg·L^−1^ (96 h)	47.2% relative activity (vs. IMT)	Dai et al. (2010)
	*C. pipiens*	IMT	LC_50_ = 0.45 mg·L^−1^ (24 h)	Strongest lethality after 24 h.	Dai et al. (2010)
	*C. pipiens*	5-hydroxy-IMT	LC_50_ = 0.68 mg·L^−1^ (24 h)	Intermediate lethality.	Dai et al. (2010)
	*C. pipiens*	Olefin-IMT	LC_50_ = 0.52 mg·L^−1^ (24 h)	Initial strong effect (2 h), but weakest after 24 h; detoxification likely.	Dai et al. (2010)
	Honeybees	IMT	LD_50_ = 0.84 mg·L^−1^ (48 h), applied via direct contact	High toxicity.	Ma et al. (2021)
Aquatic organism	*Danio rerio*	IMT	LC_50_ >100 mg·L^−1^ (96 h); Exposure to IMT aqueous solutions at various concentrations under static/semi-static regimes.	Low toxicity.	Ma et al. (2021)
	*Daphnid magna*	IMT	EC_50_ 12.80 mg·L^−1^ (48 h); Exposure to IMT aqueous solutions at various concentrations.	Low toxicity.	Ma et al. (2021)
Mammals/human	Rat	IMT	Oral administration LD_50_ 328.66 mg/kg	Rapid systemic distribution (peak at 4 h); exhibits moderate toxicity in rats.	Zhuang et al. (2024)
	Rat	Hydroxyl-IMT	Oral administration LD_50_ 357.17 mg/kg	Exhibits moderate toxicity in rats.	Zhuang et al. (2024)
	Rat	Nitroso-IMT	Oral administration LD_50_ 251.12 mg/kg	Exhibits moderate toxicity in rats.	Zhuang et al. (2024)
	Rat	CMDIA	Oral administration LD_50_ 395.38 mg/kg	Exhibits moderate toxicity in rats.	Zhuang et al. (2024)
	Rat	CMIO	Oral administration LD_50_ 171.78 mg/kg	Exhibits moderate toxicity in rats.	Zhuang et al. (2024)
	Rat	M243	Oral administration LD_50_ 3588.87 mg/kg	Exhibits low toxicity in rats.	Zhuang et al. (2024)
	Human neural stem cells	IMT	100 nM-1 μM IMT was directly added to the cell culture medium.	Disrupted NSC maintenance/differentiation; neural tube defect risks; shows pronounced toxicity.	Li et al. (2024)
	Newborns (prenatal)	IMT	Analysis of neonatal first urine; median concentrations 0.024–0.291 ng/mL (urine)	93% detection rate; IMT linked to preterm birth (odds ratio = 3.24); confirms maternal-fetal transfer	Tang et al. (2024)

Regarding its metabolites, 5-hydroxy-IMT generally showed lower toxicity compared to the parent compound. However, its olefin derivative displayed markedly increased acute toxicity to green algae (EC₅₀ = 1.94 mg L^−1^) (Yin et al., 2025), indicating that structural transformation may enhance toxicity toward primary producers. Another major metabolite, CMDIA, exhibited an overall toxicity profile comparable to that of IMT. In contrast, CMIO showed significantly reduced toxicity to both fish and *D. magna*—classified as “non-toxic”—while its toxicity to green algae was slightly elevated. This discrepancy may be attributed to alterations in functional groups (e.g., transformation of –NH–NO₂ to –NH₂), which potentially diminish neurotoxicity but enhance the inhibition of photosynthesis and other metabolic processes (Yin et al., 2025).

### 4.2 Ecotoxicity to soil organisms

For soil organisms, this study presents a comprehensive evaluation of sublethal effects of IMT on *Eisenia fetida*, a key terrestrial bioindicator. Earthworms were exposed to IMT-treated soil (0.3 and 1.0 mg·kg^−1^) for 28 days, followed by a 28-day recovery in uncontaminated soil (Table 2; Zhang et al., 2017). IMT significantly inhibited glutathione S-transferase (GST), carboxylesterase (CE), catalase (CAT), and peroxidase (POD) activities, indicating oxidative stress, while superoxide dismutase (SOD) remained unaffected. Upon transfer to clean soil, GST, CE, CAT, and SOD activities recovered, some exceeding control levels, suggesting physiological adaptation. However, POD activity at 0.3 mg·kg^−1^ remained suppressed through day 35. Comet assay revealed significant DNA damage, with elevated Olive Tail Moment (OTM) values during exposure and partial normalization during recovery, implying limited genotoxic repair. Overall, IMT induces oxidative and genotoxic stress in *E. fetida*, though partial recovery is possible post-exposure (Table 2; Zhang et al., 2017).

Beyond soil ecotoxicity, IMT’s photochemical properties and its photo-induced toxicity also warrant attention (Fan et al., 2022). Compared to acetamiprid, TMX, and IMI, IMT exhibits notably stronger photo-enhanced toxicity against *Vibrio fischeri* under standard illumination. Photodegradation follows primarily direct photolysis with a rate constant of 6.48 × 10^−3^ min^−1^, indicating rapid transformation into intermediates (Fan et al., 2023). These photoproducts chiefly drive the enhanced toxicity, as evidenced by significant mitigation upon addition of reactive oxygen species (ROS) scavengers, underscoring ROS’s catalytic role in photochemical reactions.

Photolytic intermediates demonstrate greater toxicity than the parent compound, a critical factor in photo-enhanced effects (Sinclair and Boxall, 2003). Moreover, dissolved organic matter (DOM) substantially influences IMT photochemistry by elevating ROS generation, though its effects vary among NEOs. In IMT’s case, ROS facilitate conversion of highly toxic intermediates to less harmful products, thus attenuating overall phototoxicity (Fan et al., 2023). Consequently, DOM and ROS scavengers modulate ROS dynamics and transformation pathways, producing compound-specific variations in photolysis rates and photo-induced toxicity.

### 4.3 Insects

IMT, a systemic NEO, exhibits high efficacy against target pests but raises substantial concerns regarding its ecological safety (Ma et al., 2021). Accumulating evidence shows that IMT is acutely toxic to pollinators, including honeybees (Dussaubat et al., 2016; Wu-Smart and Spivak, 2016), with broader ecosystem-level implications (Beketov et al., 2013). IMT had high toxicity to *Apis mellifera* (48 h LD_50_ = 0.84 mg·L^−1^) (Table 2) (Ma et al., 2021). Its major metabolites—5-hydroxy-IMT and olefin-IMT—retain bioactivity against *Aphis craccivora* and *Culex pipiens*, but display markedly reduced toxicity toward *Nilaparvata lugens* (Table 2) (Dai et al., 2010), suggesting metabolic transformation can attenuate insecticidal potency. Moreover, IMT and its derivatives have been reported to exert toxicity on various non-target terrestrial organisms, including the parasitic was *Binodoxys communis* and the soil indicator earthworm *E. fetida* (Du et al., 2023; Zhang et al., 2017).

IMT is under consideration for integrated pest management in cotton systems; however, sublethal exposure (LC₁₀: 11.48 mg·L^−1^; LC₃₀: 28.03 mg·L^−1^) significantly compromises survival, parasitism, and longevity of the parasitoid *B. communis* (Table 2), while extending developmental duration. Notably, recent findings reveal that IMT induces transgenerational effects, with offspring inheriting stress phenotypes. Multi-omics analyses show that IMT disrupts host energy metabolism and detoxification pathways, accompanied by significant alterations in gut microbial composition and gene expression (Du et al., 2023), implying microbiome-mediated modulation of physiological stress.

Systematic toxicity assays confirm potent activity of IMT and its two major metabolites—5-hydroxy-IMT and olefin-IMT—against *A. craccivora* (LC₅₀ < 1 mg·L^−1^). Against *N. lugens*, IMT demonstrates strong efficacy (LC₅₀ = 0.77 mg·L^−1^), while olefin-IMT exhibits only ~47% relative toxicity (LC₅₀ = 1.63 mg·L^−1^), and 5-hydroxy-IMT shows negligible effect (LC₅₀ > 4 mg·L^−1^) (Table 2) (Gorman et al., 2008; Wang et al., 2008, 2009). These results suggest that hydroxylation and olefin formation during metabolism may attenuate IMT’s insecticidal potency, particularly the 5-hydroxylation metabolite which significantly reduces toxicity against *N. lugens*, potentially contributing to resistance development. These findings suggest that metabolic hydroxylation may facilitate resistance development via detoxification. In *C. pipiens* larvae, all three compounds elicited acute toxicity within 2 h; however, IMT showed the highest lethality at 24 h, followed by olefin- and 5-hydroxy-IMT, reflecting distinct toxicokinetic profiles (Dai et al., 2010). Collectively, IMT and its metabolites exert multifaceted effects across trophic levels, involving neurotoxicity, enzymatic disruption, microbiome shifts, and heritable stress. Future pesticide risk assessments must integrate the sublethal effects and long-term exposure risks of these parent compounds and their metabolites, with particular attention to the potential ecological risks arising from mixed exposure to NEOs and their metabolites (Zhuang et al., 2024).

### 4.4 Toxicity and developmental risks in mammals centered on IMT

Emerging evidence links chronic NEO exposure to neurodevelopmental disorders in children and impaired lung function in adults (Xu et al., 2021; Tu et al., 2023). The unique neurotoxicity of NEOs raises concerns for neonates (Tang et al., 2024), yet systematic studies on prenatal exposure effects remain limited. A recent biomonitoring study detected 13 NEOs and metabolites in first-void urine from 92 newborns, with over 93% positivity, confirming maternal-fetal transfer. Notably, N-desmethyl-IMI, IMI, TMX, and sulfoxaflor showed the highest detection rates (59–87%) and median concentrations (0.024–0.291 ng/mL). Regression analysis linked higher IMT exposure to increased preterm birth risk (odds ratio = 3.24; Table 2), implicating in utero IMT as a risk factor for adverse outcomes (Tang et al., 2024). Tang et al. (2024) reported a positive association between detectable IMT levels in newborn urine and preterm birth risk; however, this observational correlation does not establish causality. The study’s moderate sample size, potential unmeasured confounding factors (e.g., maternal health, diet, socioeconomic status), and concurrent neonatal exposure to multiple NEOs and other environmental chemicals limit the strength of inference and indicate that the findings should be viewed as a preliminary signal requiring confirmation in larger, better-controlled cohorts.

In mice, IMT exhibited rapid systemic distribution post-oral administration, peaking at 4 h without tissue accumulation. Seven IMT metabolites were preliminarily identified in rat, including hydroxyl IMT (M277) and M292 (hydroxylation), CMDIA and M214 (nitrate ester hydrolysis), M243 (methylation), CMIO (urea formation), and nitroso IMT (nitro group reduction). These transformations primarily targeted the electronegative N-nitro group (–N–NO₂), which underwent sequential reduction to nitroso (–N–N=O), amine (–NH₂), and finally hydrolysis to urea (Zhuang et al., 2024). With the exception of M243, which showed low toxicity in rats, the other six IMT metabolites showed all but moderate toxicity (Zhuang et al., 2024). These findings elucidate IMT’s mammalian fate and inform agricultural safety.

Developmental neurotoxicity was assessed using human neural stem cells (NSCs) and neuronal cell lines exposed to 19 common pollutants, including NEOs, at environmental concentrations (100 nM, 1 μM). NSCs were most sensitive, with IMT showing pronounced toxicity (Table 2) (Li et al., 2024). Prenatal NEO exposure correlates with neurodevelopmental deficits and poorer neurological outcomes in newborns (Cimino et al., 2017; Pan et al., 2022; Choi et al., 2021; Liu et al., 2021). Collectively, these data highlight that novel NEOs, particularly IMT, may disrupt NSC maintenance and differentiation, increasing risks of neural tube defects and developmental disorders. This underscores the critical need for long-term toxicological monitoring and neurotoxicity assessment in vulnerable populations. Although the exposure levels examined exceed typical population biomonitoring estimates, they remain relevant for modeling peak-exposure scenarios and accounting for the heightened vulnerability of developing neural tissues. Such concentrations are also intentionally used to elicit measurable phenotypic responses in controlled systems, aiding the identification of hazards and mechanistic pathways that warrant further investigation at more physiologically realistic doses.

### 4.5 Residue risk and human exposure of IMT in the agricultural environment and food chain

The extensive use of IMT in modern agriculture has resulted in its widespread occurrence in environmental matrices and food chains, raising concerns regarding chronic human exposure (Alsafran et al., 2022). In China, intensive maize and rice cultivation has been identified as a major source of IMT input into agroecosystems (Liao et al., 2024). Residues of IMT are frequently detected in soils and edible crops; for example, in leafy vegetables such as pak choi, the half-lives of six pesticides including IMT range from 2.2 to 12 days, yet terminal residues can remain substantial under open-field conditions (Tang et al., 2021). Environmental persistence of IMT is further supported by regional surveys. In the Pearl River Delta, IMT and related NEOs were detected in 95% of agricultural soil samples, with the highest median concentrations (23 ng/g) observed in intensively managed vegetable-growing soils, reflecting repeated application practices and limited degradation under field conditions (Yu et al., 2021).

Dietary intake represents the primary exposure pathway for the general population. Food monitoring studies indicate that IMT can be efficiently transferred through the food chain. A nationwide survey of commercial milk samples detected at least one NEO or its metabolite in all samples, with IMT frequently present as a component of multi-residue mixtures, showing detection frequencies ranging from 50 to 88% (Wei et al., 2023). Similarly, dietary exposure assessments in children revealed ubiquitous detection of IMT in fruits and vegetables, often co-occurring with several other NEO compounds (Zhang et al., 2019). These findings indicate significantly heightened risks of IMT exposure among children.

In summary, the human health risk associated with IMT arises from the convergence of its environmental persistence, intrinsic neurotoxicity, and confirmed dietary exposure. While the long-term health consequences of chronic low-dose IMT exposure remain incompletely characterized, the consistent detection of IMT in staple foods, including those consumed by children, warrants precautionary management. Therefore, IMT-specific multi-source exposure assessment and targeted regulatory frameworks are urgently needed to mitigate potential risks to human health.

## 5 Conclusion and prospects

IMT exemplifies the complex environmental challenges posed by fourth-generation NEO. This review provides the first comprehensive synthesis of its environmental fate, microbial degradation pathways, and cross-species toxicological effects. The frequent detection of IMT and its metabolites across diverse ecological zones and biological systems—including humans—highlights the urgent need for integrated, metabolite-centered ecological risk assessments. To ensure the protection of environmental and human health, regulatory frameworks must be modernized to address the persistence, transformation product toxicity, and cumulative exposure risks associated with NEO use.

Current environmental fate studies of IMT emphasize its persistence and widespread detection in soil, water, and agricultural products. However, gaps remain in understanding its long-range transport potential, atmospheric behavior, and interactions with co-occurring agrochemicals. Investigations incorporating fate modeling under varied climatic and land-use scenarios are essential to predict IMT’s dispersion across agroecosystems, urban landscapes, and distant biomes. Such efforts would support the development of geographically adaptive risk mitigation strategies. Although microbial degradation has been proposed as a sustainable remediation approach, existing studies predominantly focus on isolated strains or enzymes. Future research should shift toward community-level and microbiome-informed frameworks that consider horizontal gene transfer, metabolic redundancy, and resilience under environmental stressors. Moreover, integrating plant–microbe interactions, such as phytoremediation-assisted degradation and rhizosphere microbiota dynamics, could amplify degradation efficiency and ecological restoration.

Pollinators (e.g., bees) are a high-risk group due to the high contact and oral toxicity of IMT, coupled with its systemic nature in flowering crops. Soil organisms (e.g., earthworms) face a lower immediate acute risk; however, the persistence of IMT in soil raises significant concerns regarding long-term chronic effects and impacts on microbial function. For aquatic organisms, particularly cladocerans, the parent compound poses a low acute risk, but its metabolites—such as the olefin derivative, which is both highly toxic to this group and frequently detected in surface waters—present a considerable ecological threat. Toxicological assessment of IMT and its metabolites should move beyond acute endpoints and model organisms. Expanding to non-model species, especially soil invertebrates, aquatic producers, and vertebrates, will reveal hidden ecotoxicity impacts across trophic levels. Advanced techniques—such as omics-based biomarkers, multi-generational assays, and *in vitro* models simulating human-relevant exposure pathways—will be instrumental in bridging the gap between lab and field observations, particularly under climate-mediated stress conditions.

A major challenge in assessing the environmental risks of IMT is that exposure rarely occurs inisolation; biomonitoring studies (Tang et al., 2024; Wei et al., 2023) consistently show that humans and wildlife encounter complex mixtures of NEOs and other agrochemicals, particularly in regions like China where multi-pesticide use is widespread. Such co-exposure raises substantial concerns about mixture toxicity, as most NEOs—including IMT—share a common mode of action as nicotinic acetylcholine receptor agonists, making concentration addition likely. Consequently, even when individual compounds are present below their no-effect thresholds, their combined levels may still produce biologically significant effects an issue not adequately addressed by current single-substance regulatory frameworks. These considerations underscore the need for future research and policy to adopt mixture-oriented assessment strategies that systematically evaluate additive and potentially synergistic interactions among IMT, co-applied NEOs, and other pesticides.

Finally, to ensure environmental sustainability and food safety, policy frameworks must evolve in step with scientific advancements. Regulatory systems should incorporate metabolite-specific toxicity, cumulative exposure risks, and vulnerable population thresholds into pesticide approval and residue limit standards. The case of IMT exemplifies the complex ecological and human health risks posed by NEOs. Addressing these challenges requires an interdisciplinary, systems-level approach that spans environmental science, toxicology, molecular biology, and public health.
